# Optimization of Long-Acting Bronchodilator Dose Ratios Using Isolated Guinea Pig Tracheal Rings for Synergistic Combination Therapy in Asthma and COPD

**DOI:** 10.3390/ph15080963

**Published:** 2022-08-03

**Authors:** Elena Menchi, Charaf El Khattabi, Stéphanie Pochet, Olivier Denis, Karim Amighi, Nathalie Wauthoz

**Affiliations:** 1 Unit of Pharmaceutics and Biopharmaceutics, Department of Pharmacotherapy and Pharmaceutics, Faculty of Pharmacy, Université libre de Bruxelles,1050 Brussels, Belgium; karim.amighi@ulb.be (K.A.); nathalie.wauthoz@ulb.be (N.W.); 2 Unit of Pharmacology, Pharmacotherapy and Pharmaceutical Care, Department of Pharmacotherapy and Pharmaceutics, Faculty of Pharmacy, Université libre de Bruxelles, 1050 Brussels, Belgium; charaf.el.khattabi@ulb.be (C.E.K.); stephanie.pochet@ulb.be (S.P.); 3 Laboratory of Immune Response, Sciensano, 1180 Brussels, Belgium; olivier.denis@sciensano.be

**Keywords:** asthma, COPD, bronchodilators, combination, synergy, ex vivo, airway relaxation

## Abstract

The co-administration of a long-acting β2-agonist (LABA), and a long-acting muscarinic antagonist (LAMA), has been shown to be beneficial in the management of non-communicable chronic respiratory diseases, such as asthma and chronic obstructive pulmonary disease (COPD). The resulting relaxation of the airways can be synergistically enhanced, reducing symptoms and optimizing lung function. This provides an insight into more effective treatments. In this study, the LABAs formoterol fumarate dihydrate (FOR) and indacaterol maleate (IND) were each associated with tiotropium bromide monohydrate (TIO) to assess their synergistic potential. This was done using an appropriate ex vivo model of isolated perfused guinea pig tracheal rings, and pharmacological models of drug interaction. Among the dose ratios studied for both types of combination, a higher synergistic potential was highlighted for FOR/TIO 2:1 (*w/w*). This was done through three steps by using multiple additions of drugs to the organ baths based on a non-constant dose ratio and then on a constant dose ratio, and by a single addition to the organ baths of specific amounts of drugs. In this way, the synergistic improvement of the relaxant effect on the airways was confirmed, providing a basis for improving therapeutic approaches in asthma and COPD. The synergy found at this dose ratio should now be confirmed on a preclinical model of asthma and COPD by assessing lung function.

## 1. Introduction

Asthma and COPD are two major chronic non-communicable respiratory diseases whose prevalence and incidence have greatly increased in last decades. According to recent estimates, they each currently affect over 350 million people worldwide. This number is likely to continue to grow due to population aging and continuous exposure to risk factors [1,2,3]. Furthermore, the annual related mortality is estimated at 495,000, and more than 3 million deaths for asthma and COPD, respectively [1,2]. The socio-economic impact associated with these diseases is significant. Respiratory diseases account for 6% of the annual European health budget. Of the total cost of respiratory disease, 28% and 56% are attributed to asthma and COPD, respectively. [2,3]. For this reason, asthma and COPD are among the World Health Organization’s priorities in the prevention and control of non-communicable diseases [1,2]. These two high-burden chronic respiratory diseases remain difficult to control for many patients. The achievement of good symptom control and the prevention of risks of adverse outcomes (i.e., exacerbations, treatment side effects, persistent airflow limitations, disease-related mortality) are two goals to reach in the management of these diseases [1,2]. If treatments containing inhaled corticosteroids are the cornerstone of asthma treatment, people who have ongoing symptoms despite good adherence and inhaler technique often need additional medications, such as bronchodilators [1]. Conversely, bronchodilators, especially long-acting bronchodilators, are indicated as a first-line treatment for COPD, and are given in combination for patients with more severe symptoms [2]. The concomitant administration of LABA with LAMA has demonstrated a synergistic effect on airway smooth muscle (ASM) relaxation [4]. Optimizing bronchodilation through distinct and complementary mechanisms of action has the potential to provide treatments that reduce drug doses and improve efficacy, effectiveness, and safety [5], as recommended by the Global Initiative for Asthma [1] and the Global Initiative for Chronic Obstructive Lung Disease [2]. Therefore, considering the pharmacological interaction between these two classes of bronchodilators might lead to a significant improvement in the medication of asthma and COPD patients.

Recent decades have seen a growing interest in developing strategies to optimize combination therapies for the treatment of obstructive respiratory diseases. The aim is to balance and reduce the doses of each bronchodilator, while maintaining an effective level of bronchodilation, for the improved safety profile of dual bronchodilator therapy [5,6]. The investigation of these criteria can be carried out early in ex vivo experimental models by pharmacological studies. The use of isolated perfused parts of the respiratory tract is a well-known technique for studying the effect of drugs on the contractile tone of the ASM [7,8,9]. Furthermore, appropriate animal models, such as guinea pig lung tissue, are useful to predict the potency and efficacy of drugs in human lung tissue. Indeed, guinea pig airways have proven to be a relevant pharmacological tool for studying bronchodilator drugs as many similarities were found regarding human receptor pharmacology [10,11,12,13]. Mediators involved in inflammation and physiological responses are similar in guinea pig and human airways, while some are inert in the airways of rats and mice [11,13,14]. In addition, the use of bronchodilator drugs has been described as clinically effective in guinea pig airways, as well as in human airways, for both asthma and COPD conditions [15].

To date, mainly the pharmacological interactions between LABAs and LAMAs in marketed fixed-dose combinations were studied. These investigations were done to explore unknown underlying mechanisms of action, and/or find the optimal dose of each drug. Most of these investigations were done in randomized controlled trials [16,17] or using ex vivo studies on isolated perfused human medium bronchi and small airways [18,19]. However, interest in generating robust preclinical data on synergistic activity between asthma and COPD drugs to develop new fixed-dose combinations is increasing [6]. Therefore, new LABA/LAMA combinations with drugs that have proven prolonged bronchodilator properties is an interesting approach for study.

TIO is the only LAMA approved for use in asthma as add-on therapy [20,21] and has been used for about two decades in COPD [22]. This drug is therefore a candidate of choice to be studied in combination with a LABA when considering both chronic respiratory diseases: asthma and COPD. Numerous clinical studies have been conducted with this drug and it has shown evidence of improved lung function. With 24 h of action, TIO provides a good safety profile and has demonstrated clinical benefit in patients when added to a corticosteroid with or without LABA in asthma or COPD [21,23,24].

For this study, FOR and IND were each associated with TIO to form dual combinations. FOR is a well-known and widely used drug that provides 12 h of action for asthmatic patients. This drug is indicated as a controller therapy. It is also highly recommended as a reliever therapy in combination with a low-dose corticosteroid, instead of a short-acting β2-agonist, with which the risk of exacerbation is higher [1]. FOR is also indicated to treat bronchial obstruction in COPD patients. This drug is already included in various types of physical mixtures (i.e., in combination with a corticosteroid, with a LAMA, or with both types of drugs) [25,26,27]. Furthermore, its use with TIO for the treatment of COPD has been investigated in clinical studies ranging from one day to several weeks. Greater improvements in lung function, as well as improved symptom scores, and reduced use of rescue medication, have been shown in comparison with monotherapies [2,28]. IND is called “Ultra-LABA” because of its long duration of action of over 24 h. This drug has recently been approved for asthmatic patients. Indeed, IND is indicated for the treatment of asthma in a once-daily dose dual combination with a corticosteroid, and a triple combination with a corticosteroid and a LAMA [29,30]. In addition, benefits in terms of the bronchodilation effect on COPD patients have been demonstrated in combination with the LAMA TIO [23].

In this context, the following study was performed to identify and characterize the type of pharmacological interaction between LABA and LAMA for the FOR/TIO and IND/TIO combinations. Their impact on the contractile tone of guinea pig tracheal rings was assessed, focusing on the dose ratio of LABA/LAMA (*w/w*) for an optimization of ASM relaxation. Ultimately, this research could serve as a basis to positively impact the treatment and management of asthma and COPD.

## 2. Results

### 2.1. Individual Relaxant Effect

**Figure 1:**
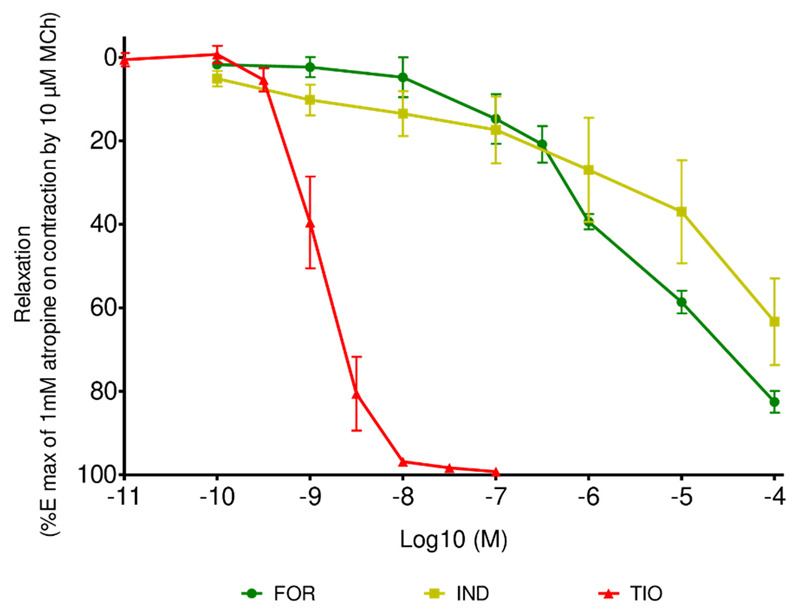
Relaxant effect of FOR, IND, and TIO administered as monocomponents. Data are expressed as mean ± SEM (*n* = 6). MCh: methacholine; Emax: maximal effect.

All studied drugs induced a concentration-dependent relaxation of the guinea pig tracheal rings, precontracted with methacholine (experimental negative logarithm of the half maximal effective concentration (pEC50) values: TIO 8.8 ± 0.3, FOR 5.4 ± 0.3, IND 5 ± 1). In the tested concentration range, TIO, but not the studied LABAs, completely suppressed the contractile tone of the tracheal rings induced by methacholine administered at the submaximal effective concentration size between 50% and 70% of the maximum concentration (EC50-70) (Figure 1). The assessment of the contractile effect of methacholine over 4 h was carried out in preliminary studies before the start of the experiment with bronchodilator drugs. A decline of the methacholine-induced airway contraction in guinea pig tracheal rings of less than 10% was observed and did not significantly affect the relaxation induced by the studied bronchodilator drugs. Indeed, the relaxant effect induced by the presence of bronchodilator drugs was significantly greater than in their absence (*p* < 0.05, ANOVA). Similarly, the vehicle had no significant effect on tracheal ring tone at the administered concentrations. The relaxant effect induced by the bronchodilator drugs was significantly greater than that of the vehicle at its higher concentration (*p* < 0.05, ANOVA) (Supplementary Material: Table S1).

### 2.2. Interaction between LABA and LAMA Based on a Non-Constant Dose Ratio

**Figure 2:**
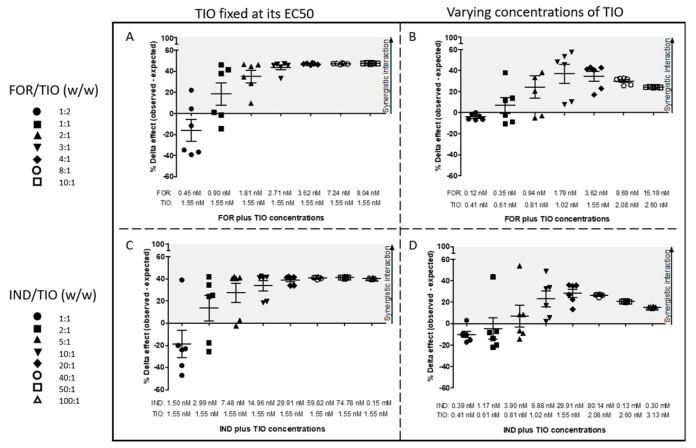
ΔE between observed and expected relaxant response induced by FOR plus TIO (**A**,**B**), and by IND plus TIO (**C**,**D**) predicted by the BI theory for the range of concentrations tested in the study. Data are expressed as mean ± SEM from experiments performed using samples from *n* = 6 different subjects. Each point represents one subject. Each symbol represents a dose ratio. BI: Bliss Independence; EC50: half maximal effective concentration.

Cumulative addition of the drugs results in a different delta effect (ΔE) graphical plot depending on the approach used to obtain the dose ratios. By combining TIO at its half maximal effective concentration value with varying concentrations of LABA added cumulatively, the difference between the observed and the expected relaxant response increased and tended to reach a plateau (Figure 2A,C). This difference reached a maximum value before decreasing as the concentrations of both drugs varied in the combination at each addition of drugs (Figure 2B,D). Both combinations provided a synergistic relaxant response in guinea pig tracheal rings according to the Bliss independence (BI) theory, but not at all dose ratios (Figure 2A–D).

Cumulative addition of FOR to a fixed concentration of TIO induced synergistic interaction with a positive 95% confidence interval from the third up to the last studied dose ratio (FOR 1.81 up to 9.04 nM, TIO 1.55 nM), and a maximal improvement of the relaxant response of +47.4 ± 0.3% compared to the expected response calculated using the BI equation (Figure 2A). Cumulative addition of varying concentrations of FOR and TIO resulted in a synergistic interaction with a positive 95% confidence interval from the fourth up to the last studied dose ratio (FOR 1.79 up to 15.19 nM, TIO 1.02 up to 2.60 nM), and a maximal improvement of the relaxant response of +34 ± 11% compared to the expected response calculated using the BI equation (Figure 2B).

The synergistic interaction obtained by cumulative addition of IND to a fixed concentration of TIO shows a positive 95% confidence interval from the fourth up to the last studied dose ratio (IND 14.96 nM up to 0.15 mM, TIO 1.55 nM), and a maximal improvement of the relaxant response of +41.2 ± 0.4% compared to the expected response calculated using the BI equation (Figure 2C). IND combined with TIO, both in varying concentrations, are synergistic with a positive 95% confidence interval from the fourth up to the last studied dose ratio (IND 9.88 nM up to 0.30 mM, TIO 1.02 up to 3.13 nM), and a maximal improvement of the relaxant response of +28 ± 9% compared to the expected response calculated using the BI equation (Figure 2D). The Unified Theory (UT) analysis confirmed that both combinations result in synergistic interaction over a wide range of concentrations for both ways of cumulative addition. The interaction magnitude increases at each addition of drug (Table 1).

The pharmacological interaction study between LABA and LAMA, based on a non-constant dose ratio, allowed the selection of dose ratios for which the pharmacological interaction was further investigated. Synergistic interaction had to be confirmed by both BI and UT methods (i.e., positive ΔE and 95% confidence interval, and combination index (CI) value lower than 1) to select these dose ratios. Moreover, only the first combined concentration providing these conditions was chosen for each LABA/LAMA combination in each approach. This was because of the cumulative nature of the addition of the different drug concentrations to the organ bath, and the time-dependency of its relaxant effect. These conditions were met for FOR/TIO combinations 1.81 nM/1.55 nM (i.e., 0.030 µg/0.015 µg) and 1.79 nM/1. 02 nM (i.e., 0.030 µg/0.010 µg), giving dose ratios of 2:1 (*w/w*) and 3:1 (*w/w*), respectively. Similarly, they were met for IND/TIO 14.96 nM/1.55 nM (i.e., 0.152 µg/0.015 µg) and 29.91 nM/1.55 nM (i.e., 0.304 µg/0.015 µg), corresponding to dose ratios 10:1 (*w/w*) and 20:1 (*w/w*), respectively.

### 2.3. Interaction between LABA and LAMA Based on a Constant Dose Ratio

**Figure 3:**
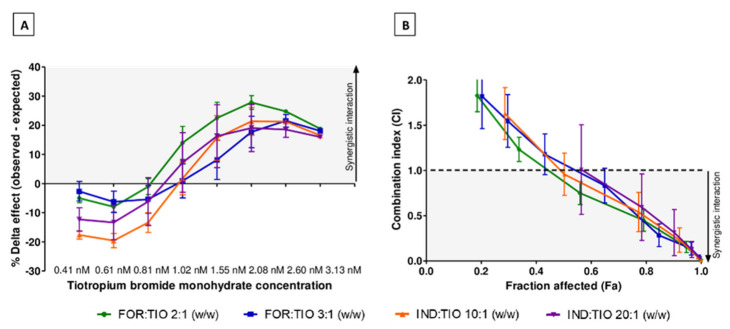
Pharmacological interaction analysis in guinea pig tracheal rings induced by FOR plus TIO (green and blue curves), and by IND plus TIO (orange and purple curves) for the range of concentrations tested in the study. Dose ratios for each curve are specified below the graphs. (**A**) ΔE value between observed and expected relaxant response predicted by the BI theory. (**B**) Combination index plot obtained according to the UT (7 data points are outside the axis limit). Data are expressed as mean ± SEM from experiments performed using samples from *n* = 5–6 different subjects. BI: Bliss independence; CI: combination index; Fa: fraction affected; UT: Unified Theory.

Amounts of drugs in the constant dose ratio combinations were defined based on TIO, which was experimentally more potent than the LABAs in guinea pig tracheal rings (i.e., its pEC50 value is higher than those of FOR and IND) (Figure 1). Consequently, the cumulative addition of this drug was similar for all combinations tested. The ΔE graphical plots resulting from the cumulative addition of combined drugs at a constant dose ratio are sigmoidal (Figure 3A). Synergistic interactions were identified for all studied combinations, but only from a certain concentration, which depended on the combination and dose ratio. FOR/TIO combination with the dose ratio 2:1 (*w/w*) showed synergistic results at lower drug concentrations, but only after three cumulative additions to the organ baths. Other studied combinations showed synergistic results after a further cumulative addition. Moreover, the maximal improvement of the relaxant response, compared to the expected response calculated using the BI equation, was defined for the FOR/TIO combination and the IND/TIO combination (FOR/TIO 2:1 (*w/w*) +27.8 ± 5.8%, FOR/TIO 3:1 (*w/w*) +21.5 ± 4.8%, IND/TIO 10:1 (*w/w*) +21.4 ± 11.7%, IND/TIO 20:1 (*w/w*) +19.1 ± 18.1%) (Figure 3A). There were no significant differences between the maximum ΔE for the studied constant dose ratio combinations (*p* > 0.05, ANOVA). The magnitude of interaction for these combinations is described by a CI plot representing the CI value in relation to the fraction affected, which is associated to the relaxant effect obtained for each studied combination (Figure 3B). This plot confirms that the pharmacological interactions were synergistic (CI below 1) beyond a defined dose that is cumulatively added. Again, the earliest synergistic interaction was found for FOR/TIO combination with the dose ratio 2:1 (*w/w*), although not significantly earlier than the other conditions tested (*p* > 0.05, ANOVA).

As TIO was added in the same way for all dose ratios tested, the value of its DRI was determined at concentrations giving a relaxant response of 50% (Table 2). These values were greater than 1 for all combinations with a slightly greater value for the FOR/TIO combination with the dose ratio 2:1 (*w/w*). Similarly, the same procedure was followed for the DRIs of the LABAs. The DRI values of FOR were used to compare both FOR/TIO combinations. Also, the DRI values of IND were used to compare the IND/TIO combinations. These values were all positive, indicating a favorable situation for dose reduction. However, dose ratios involving lower LABA concentrations were characterized by higher DRI values (data not shown).

The aim was to determine an appropriate dose ratio of LABA/LAMA combinations resulting in synergistic airway relaxation. Therefore, the dose ratio showing strong synergy and leading to a high dose reduction value when compared with the same drugs in monotherapy, was chosen to be further studied.

### 2.4. Interaction between LABA and LAMA Over Time

**Figure 4:**
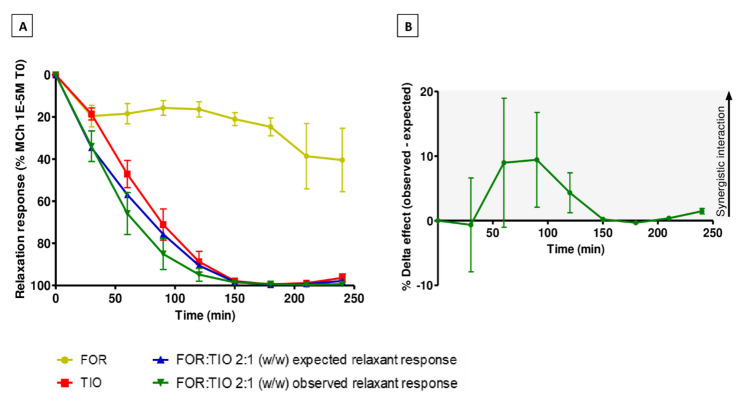
Pharmacological interaction analysis in guinea pig tracheal rings induced by defined amounts of FOR and TIO in a 2:1 (*w/w*) dose ratio. (**A**) FOR (yellow curve), TIO (red curve), observed (green curve), and expected (blue curve) relaxant responses over time. (**B**) ΔE value between observed and expected relaxant response over time predicted by the BI theory. Data are expressed as mean ± SEM from experiments performed using samples from *n* = 5–6 different subjects. BI: Bliss independence.

Based on the results obtained in the interaction study using a constant dose ratio, the relaxation and ΔE value of the FOR/TIO combination with the dose ratio 2:1 (*w/w*) with defined amounts of drugs were assessed over time (Figure 4). Amounts of drugs giving the maximal improvement of the ΔE in the previous study (FOR 0.04 µg, and TIO 0.02 µg, which corresponds to a concentration of 2.42 nM and 2.08 nM, respectively) were used for addition to the organ baths at the starting time. The observed relaxation curve shifts slightly to the left compared to the expected one (Figure 4A), although these two curves are still similar for the first 30 min of the experiment. Furthermore, the calculation of the ΔE value at each time point resulted in an assessment of the pharmacological interaction over time, according to the BI theory (Figure 4B). This interaction became synergistic after 30–60 min of contact time between the combined drugs and the tracheal rings. The maximal ΔE value was obtained after 90 min of tracheal exposure to the combination of drugs (FOR/TIO 2:1 (*w/w*) +9.4 ± 0.2%), before decreasing when approaching the maximal relaxant response.

## 3. Discussion

The contraction of the ASM was achieved using methacholine by activation of M3-muscarinic receptors, located in the cell membrane, and associated with signal transduction pathways and ion channels. More generally, the main mechanism of contraction involves activation of phospholipase C, which is stimulated after binding of the agonist to its receptor. This protein cleaves phosphatidylinositol 4,5-biphosphate, leading to the formation of diacylglycerol and inositol 1,4,5-trisphosphate. Both second messengers generate an increase in intracellular calcium levels, leading to ASM contraction. Conversely, the addition of bronchodilator drugs to organ baths stimulated relaxation of the ASM. This is primarily mediated by the stimulation of adenylyl cyclase-coupled receptors involving an increase in intracellular signaling elements, such as the second messenger adenosine 3,5-cyclic monophosphate. This results in a decrease in intracellular calcium levels. Relaxation is also mediated by other mechanisms, such as the guanosine 3,5-cyclic monophosphate pathway and K+ channel openers. Therefore, the ASM tone is affected by a complex crosstalk between these contraction and relaxation pathways at different levels in ASM cells [31,32]. The data generated by the addition of drugs in a cumulative dose fashion highlights the impact of the potency of drugs on the precontracted guinea pig tracheal rings (Figure 1). It can be assumed that the high potency of TIO is due to its competitive behavior against the contractile drug methacholine. As a structural analogue of acetylcholine, methacholine binds directly to the predominant M3-muscarinic receptors on the ASM, inducing its contraction [33]. TIO suppresses the ASM contraction by a prolonged blockage of these receptors through a competitive antagonistic effect [34,35,36]. In contrast, β2-agonists bind to β2-adrenergic receptors, leading to ASM relaxation through a different signaling pathway. They elicit a direct bronchodilation through a subsequent decrease in intracellular calcium, as a result of protein-mediated signaling pathway by binding to β2-adrenergic receptors on the ASM [32,37,38]. They also interfere with cholinergic neurotransmission by functional antagonism by binding to prejunctional β2-adrenergic receptors [32,37,38,39,40]. This results in an inhibition of the contraction effect induced by methacholine. Moreover, studies on guinea pig tracheal smooth muscle preparations have showed that the sensitivity of ASM to β2-agonists depends on the initial contractile level of the muscle tone, and on the type of drug used to induce the contraction [41,42]. Therefore, methacholine appears to be an appropriate contractile agent. Its methyl group on the β-carbon of choline gives it a longer duration of action, thanks to its lower rate of hydrolysis and increased selectivity of action (i.e., it acts directly on muscarinic receptors and has a weak effect on nicotinic receptors) compared to acetylcholine, while maintaining very similar properties [33]. The use of other contractile agents, such as carbachol (another cholinergic agonist) and histamine, were also tested as preliminary investigations. Carbachol showed similar contractile results than methacholine. Histamine was not suitable because its contractile effect on tracheal rings was not maintained over time. Therefore, the assessment of the relaxing effect of the studied bronchodilators was not applicable. In addition, the use of methacholine to induce bronchial hyperreactivity in preclinical studies makes it a suitable contractile drug model for studying the effect of bronchodilator drugs [36]. Moreover, the concentration of methacholine inducing the initial contractile level at the EC50-70 was previously adapted to the experiments on tracheal rings by preliminary studies. Therefore, methacholine was used as a contractile agent of choice in this work.

As shown by the data obtained with both types of combination tested, combining FOR or IND with TIO leads to a significant and potent relaxation of guinea pig tracheal rings. Therefore, the concomitant administration of these drugs to suppress the contractile tone appears to be an attractive strategy to optimize the relaxation of the tracheal rings and, more broadly, the relaxation of the airways [18,43]. Experimental results also showed that a synergistic enhancement of the relaxation can be reached using lower concentrations for each drug than in monotherapies. This was also reported by another research group studying dual combinations of long-acting bronchodilators currently approved by the FDA and EMA (i.e., umeclidinium/vilanterol, aclidinium bromide/formoterol fumarate, glycopyrronium bromide/indacaterol fumarate, tiotropium bromide/olodaterol) [18,44]. However, the graphical plots representing the BI prediction of synergy (Figure 2) suggest that the dose ratio between drugs must be balanced to optimize synergy. According to the experimental conditions applied, the resulting level of tracheal tone relaxation depends on the concentration of drugs and their incubation time with the organ [45]. The greater the concentration and the longer the incubation time, the greater the level of relaxation. In addition, a cumulative drug-addition approach with a standardized incubation time for each level of concentration allows investigation of a wide range of concentrations and dose ratios. Nevertheless, a slight modification in the relaxant response is expected when a given dose ratio is tested in a single dose of each compound to the organ bath [9]. Therefore, drug concentration, drug incubation time, and cumulative drug-addition approach have a major influence on the interpretation of the results. This is illustrated by the results shown in Table 1. A distinct drug dose addition impacts the resulting relaxant effect, but could help to identify interesting dose ratios in a dual and complementary manner. As previously mentioned, only the first dose ratios resulting in a synergistic interaction according to the BI and UT analysis were selected for further investigation. This was done to minimize the influence of the doses of drugs previously added to the organ baths. Moreover, the increasing amounts of drugs over time could explain the increasing magnitude of interaction obtained using the UT analysis. Indeed, the fraction affected, which represents the fraction of inhibited contractile tone [46], continued to increase with the increasing amounts of drugs added cumulatively to the organ baths. Furthermore, Figure 3 and Figure 4 showed that low doses of LABA and LAMA resulted in negative ΔE values and positive CI values. Basically, it means antagonistic interaction between drugs. However, these results have to be interpreted with caution in this case. Indeed, the effect on the tracheal tone was not significantly different between the control condition and the tested drugs at the beginning of the experiment (*p* > 0.05, ANOVA). This is probably due to the defined experimental conditions. This could be misinterpreted by mathematical models and lead to a misinterpretation of the graphical plots for these low doses of drugs.

To date, FOR/TIO and IND/TIO combinations have only been investigated in clinical trials on COPD patients, using the same doses as in monotherapies (i.e., 12 µg, 150 µg, and 18 µg for FOR, IND, and TIO, respectively). The benefit of these combinations was concluded from the significant improvement in the clinical outcomes, but without attempting to optimize them by changing the dose ratio between the LABA and the LAMA [28,47,48]. Additivity, rather than synergy, is expected with these doses according to the experimental conditions applied.

The constant dose ratio analysis for combinations FOR/TIO 2:1 (*w/w*), FOR/TIO 3:1 (*w/w*), IND/TIO 10:1 (*w/w*), and IND/TIO 20:1 (*w/w*) was based on a similar setup, with a standardized incubation time and fixed dose ratios. For the FOR/TIO combinations, the maximum ΔE value was obtained with the 2:1 (*w/w*) dose ratio, while the dose ratio showing a higher ΔE value with IND/TIO was 10:1 (*w/w*) (Figure 3A). Again, the slight differences when comparing the same LABA/LAMA combination type show that promoting a combination with lower concentrations could lead to stronger synergistic interactions, as also documented by certain authors reporting this finding as a class effect [18,44]. As the maximum ΔE and CI values are not significantly different from one combination to another, the DRI values calculated by the computer software were considered at concentrations giving 50% of relaxation. These values indicated a favorable situation for dose reduction for all dose ratios studied (Table 2). The DRI value is independent of the pharmacological interaction type, but the higher this value, the higher the dose reduction for a defined therapeutic effect [46]. Therefore, the assessment of this value can be considered as an additional parameter for the selection of the dose ratio to be further investigated. Indeed, the selection of the combination with a dose ratio characterized by a high DRI value allows greater ease in adjusting the concentration of each drug while maintaining the therapeutic effect with improved clinical safety [46]. Under the experimental conditions, combinations with dose ratios involving lower LABA concentrations were characterized by higher DRI values for LABAs, whereas DRI values for TIO were similar when comparing the four combinations.

**Figure 5:**
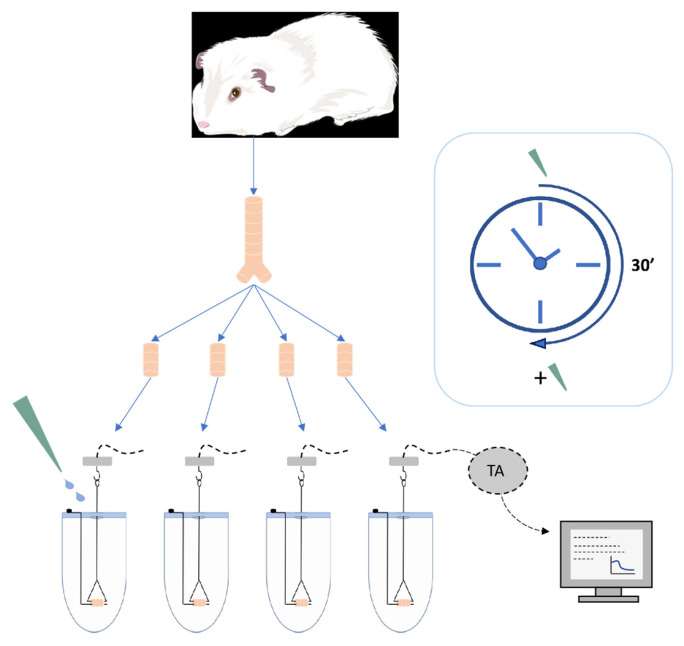
Schematic representation of the preparation of the tracheal rings and the setup used to measure ASM relaxation with a cumulative drug-addition approach. TA = transducer amplifier.

The interaction study over time on the selected dose ratio FOR/TIO 2:1 (*w/w*) was performed as a complementary study. The distinct profiles of FOR and TIO, in terms of onset and duration of action, makes them complementary, and interesting to combine. Indeed, FOR provides a faster onset of action than TIO, which is characterized by a longer duration of action over 24 h. Thus, FOR adds a greater initial peak effect, and TIO provides prolonged bronchodilation [49]. Significant additive effects of a once-daily FOR/TIO (12 µg/18 µg) treatment and its safety were demonstrated in moderate-to-severe COPD patients [50]. Therefore, a rationale must be found to obtain a well-balanced dose ratio between both bronchodilator drugs, allowing them to interact synergistically with a 24 h prolonged effect. The interaction study over time allowed the synergy to be confirmed and the selection of the appropriate amounts of drugs to be refined, without the influence of previous cumulative drug additions on the resulting relaxant response. This leads to a change in the resulting relaxation for both the individual curves and the combinations, as shown in Figure 5. At the same time, it allowed assessment of the airway relaxant effect for a few hours. This provides an insight into the effect generated by specific doses of the combined drugs, especially the onset and duration of the synergistic action resulting from this fixed-dose ratio combination over a few hours. However, the type of pharmacological interaction between drugs evolves when using the BI theory as a pharmacological model. Indeed, synergy seems to be achieved transiently over time. This is due to the maximal reachable relaxant response, which cannot be higher than 100% [4]. The ΔE value becomes smaller when the observed and expected relaxant responses are progressively closer. This value seems to be zero after 150 min of experiment, meaning Bliss independence and not synergy, according to the BI theory (Figure 4B). The relaxant effect of TIO alone also reaches the maximal relaxant response at that time (Figure 4A). Therefore, the pharmacological model is unable to discern the type of interaction resulting from the effect of both drugs beyond 150 min, because of this maximal relaxant response. Separately, the UT has not been applied in this study because it requires the acquisition of data from several drug concentrations, which does not fit the framework of this experiment. Only BI theory can be applied to single combination points experiments [4].

A major difference observed between both pharmacological models is the increasing magnitude of synergy according to the UT analysis, while the ΔE value tends to reach a maximum before decreasing in the BI theory. This latter observation must be set against the mathematical models to analyze pharmacological interactions. Indeed, each pharmacological model has its own advantages and limitations [4,51,52,53,54]. The UT analysis requires the knowledge of individual and combined concentration-relaxation curves requiring multiple data points. Therefore, large amounts of data are needed to estimate concentration-relaxation curves due to biological variation and the randomness of the experimental data [4,51,55]. Furthermore, while the CI values are closely related to the amounts of drugs added to the organ baths [46], the decrease in the ΔE value after reaching its maximum illustrates the probabilistic nature of the BI theory [51,55]. The latter assumes the independence of the action of drugs through their sites, and mechanisms of action to contribute to a common resulting effect [4,51]. However, the pathways underlying long-acting bronchodilators are not yet fully understood [32]. Therefore, there can currently be no single reference for the assessment of pharmacological interactions, especially since both BI and UT analyses would provide close and plausible results [5]. Hence the interest in highlighting synergy through several pharmacological models [51].

The resulting data must also be set against the experimental model of guinea pig tracheal rings. Indeed, regarding the ex vivo model, relaxant responses given by the tracheal rings are dependent on a standardized protocol, allowing a precise control of experimental variables with a direct and specific focus on the contractile function of the ASM [56]. If more restricted models such as lung cell cultures [57] or precision-cut lung slices [13] could be used for pharmacological interaction studies on bronchodilation, it would also be possible to identify these interactions on whole organisms. Furthermore, additional investigations assessing changes in intracellular signaling elements (e.g., ELISA assays to quantify intracellular signaling elements from bath supernatants, assessment of changes in intracellular calcium) would help to better understand which mechanisms are involved in synergistic interactions observed in this work [18]. Regarding the choice of animal species, unpublished preliminary experimental results obtained using Wistar rat tracheal rings with the same drugs, allowed a comparison to be made between guinea pig and rat animal models. Potency and interaction studies based on a non-constant dose ratio were carried out on rats by applying similar experimental conditions. Resulting data from combination studies implied molar concentrations of the LABAs up to more than one thousand times higher than the molar concentration of TIO. Likewise, excessively high dose ratios were needed to reach synergy. This provides an additional rationale for choosing the guinea pig as an ex vivo model. In addition, receptor agonists and antagonists show different potency and efficacy in rats, but also in mice, compared to humans [11]. Similarly, the beta-adrenergic receptor subtypes involved in ASM relaxation differ widely from those in the human airways, which is not the case for the guinea pig [11]. More generally, the guinea pig is an animal species with high predictive value for the assessment of drug effects in the treatment of asthma and COPD [10,11,13].

Continuing from this three-step study, the findings on isolated perfused guinea pig tracheal rings must be confirmed on preclinical models of asthma and COPD by assessing lung function before potentially proceeding to clinical trials.

## 4. Materials and Methods

### 4.1. Drugs

FOR, IND, and TIO were obtained from CHEMO Industriale Chimica s.r.l. (Saronno (VA), Italy). Methacholine was purchased from Sigma-Aldrich (St. Louis, MO, USA), and atropine sulfate from Merck (Darmstadt, Germany). Dimethyl sulfoxide was purchased from Carl Roth GmbH + Co., KG (Karlsruhe, Germany). All components used for the preparation of the Krebs-Henseleit solution (i.e., KCl, NaCl, NaHCO_3_, MgSO_4_, KH_2_SO_4_, CaCl_2_, and glucose) were purchased from VWR (Radnor, Pennsylvania, USA). Ultrapure water was obtained from a Purelab-Ultra system (Elga LabWater, Lane End, UK).

Solutions of bronchodilators were prepared daily using dimethyl sulfoxide for the stock solution and were diluted in ultrapure water. Solutions of methacholine and atropine sulfate were prepared in ultrapure water.

### 4.2. Animals

Male Dunkin Hartley guinea pigs (body weight 250–500 g) were obtained from Charles River Laboratories and housed in groups of four or five at the facilities of Sciensano (Belgian institute of public health). They received dry food (Carfil Quality, Oud-Turnhout, Belgium) and water ab libitum. All animal procedures were conducted in accordance with EU Directive 2010/63/EU for animal experiments.

### 4.3. Preparation of Isolated Tracheal Rings from Dunkin Hartley Guinea Pig

Guinea pigs were euthanized using increasing concentrations of carbon dioxide [58]. The trachea was removed and immediately placed in a Krebs-Henseleit solution with the following composition (in mM): NaCl 118.1, KCl 4.7, MgSO_4_·7H_2_O 1.2, KH_2_PO_4_ 1.2, CaCl_2_ 2.5, NaHCO_3_ 25, and glucose 5 [59,60]. After removing surrounding tissues, the trachea was cut into 3–5 mm long rings. The rings were mounted on metal rods under a 1.5 g resting tension in organ baths containing 20 mL of Krebs-Henseleit solution gassed gently with 95% O_2_, 5% CO_2_ and maintained at 37 °C [31]. All dissecting procedures were performed with extreme care to protect the internal surface from inadvertent damage. Tracheal isometric tension was determined by the organ bath technique [7,9]. Changes in isometric forces were measured using force displacement transducers and continuously recorded with a transducer amplifier and a IOX computer software, version 1.8.3.25 (EMKA Technologies, Paris, France) [60].

Equilibration of the bath conditions was carried out for at least 1 h before the experimental stimulations. The Krebs-Henseleit solution in the baths was replaced with fresh Krebs-Henseleit solution every 20 min during the hour of equilibration [31]. Following equilibration, the tracheal rings were exposed to 60 mM potassium chloride to ensure their viability [61]. Organ baths were rinsed several times to restore the resting tension, before stimulating the tracheal rings with the tested drugs.

### 4.4. Experimental Protocol

This study is part of an applied and translational research context. It is a first stage of a larger work, including the formulation of several dry powders for inhalation and their in vitro and in vivo characterization. Indeed, the main objective of this study is the selection of a dose ratio among several having shown an interesting potential to be further developed and tested in vivo on whole organism. This study is divided into three main steps in terms of added doses. Each experiment may therefore include a few or even a single dose ratio highlighted in the previous step. Indeed, at the end of each experiment, the selection of the dose ratio to be investigated could be multiple. Selecting a limited or even unique number of dose ratios is a choice that is in line with the objective of limiting the number of animal sacrifices at this stage of the work, and focusing the subsequent development of the formulation with a specific dose ratio. Therefore, it is possible that other dose ratios could be further investigated in the future.

Moreover, these experiments were carried out over a limited time of four hours. The long duration of action of the drugs cannot, therefore, be characterized in the present context. This will be done in future in vivo studies in order to establish whether the observations reported apply to a chronic administration scheme.

#### 4.4.1. Cumulative Dose Fashion

A previously defined concentration of methacholine (10 µM) was added to the organ baths to obtain a stable EC50-70 [62,63]. The assessment of tracheal relaxation was carried out on precontracted tracheal rings by multiple additions of individual or combined drugs in a cumulative dose fashion to generate semi-logarithmic concentration-relaxation curves [15]. A time interval of 30 min between concentrations was applied to reach a stable level of relaxation before the next concentration [4,64]. Cumulative concentrations of the vehicle dimethyl sulfoxide were also administered as control. At the end of the experiment, 1 µM of atropine sulfate was added to the organ baths as full agonist to achieve the maximal relaxant response for each tracheal ring, allowing calculation of a normalized relaxation percentage [65]. The contractile relaxation of tracheal rings was expressed as a percentage of the maximal relaxant response induced by atropine sulfate (1 μM) on the submaximal contractile tone induced by methacholine administered at EC50-70. The pEC50 was used to express the relaxant potency for each drug [15,66].

#### 4.4.2. Study Design

This study included two combinations, FOR/TIO and IND/TIO. Both combinations generated concentration-relaxation curves that provided data through three major steps, allowing comparison of results within and between combinations at each step. This approach leads to a refinement of the dose ratios to ultimately highlight the combination with the dose ratio that appears to promote the best airway relaxation among the dose ratios tested.

##### Interaction between LABA and LAMA Based on a Non-Constant Dose Ratio

Several dose ratios were studied for both combinations. The amounts of drugs administered in the organ bath were defined considering (i) the concentration-relaxation curves obtained for each drug alone, and (ii) the doses delivered by commercially available monotherapies. The definition used the “Direct approach” methodology for the conversion factor for the animal species chosen for the experiment [67]. The dose ratio was modified after each addition of combined drugs to the organ bath. Moreover, the dose ratios were added cumulatively to the organ bath in two ways for each combination to screen the dose ratios. These ways were either by fixing the most potent drug of the combination, i.e., the drug with the highest pEC50 value, at its half maximal effective concentration value with the only variation being the concentration of the combined drug; or by varying the concentrations of both drugs. In the latter approach, the drug with the highest pEC50 value was also used to define the concentrations added to the organ bath. Duplicating the approach regarding how to cumulatively add the drugs is primarily to demonstrate their influence on the results obtained, but also to refine the selection of the dose ratios selected for the next experiment.

##### Interaction between LABA and LAMA Based on a Constant Dose Ratio

For both studied combinations, two dose ratios were selected based on the results provided by the concentration-relaxation curves from the non-constant dose ratio interaction study. New concentration-relaxation curves were generated for both combinations at a constant dose ratio by varying only the amounts of drugs after each addition of combined drugs to the organ bath.

##### Interaction between LABA and LAMA Over Time

The evolution of the pharmacological interaction over time was assessed for a given dose ratio at defined amounts of drugs. The latter was selected based on the results provided by the concentration-relaxation curves from the constant dose ratio interaction study. These combined drugs were added to the organ bath at the starting time, and the relaxation was assessed every thirty minutes over 4 h.

### 4.5. Drug Interaction Analysis

There are numerous pharmacological models for the analysis of drug interaction. At present, there is no consensus on a single equation describing the dose-response law for quantifying the pharmacological effect [4,51,52,54]. Therefore, two common pharmacological models were used to investigate the drug interaction between LABAs and LAMAs: the BI theory and UT. This enables the identification of the synergy using an accurate statistical analysis from the BI theory, and the quantification of the magnitude of interaction using the UT analysis [4]. The assumption of the BI theory is that the action of the different drugs is independent in terms of their mode and site of action, but leads to a common outcome. This model for two drugs is represented by the following equation (1):

(1) E(x,y)= Ex +Ey −(Ex∗Ey)

where E is the fractional effect (between 0 and 1), and x and y are the concentrations of the drugs in the combination. The ΔE, which is the difference between the experimentally observed effect and the expected effect, determines the type of pharmacological interaction. A positive ΔE (i.e., an observed effect higher than the expected effect) indicates a synergistic pharmacological interaction. A negative ΔE implies an antagonist pharmacological interaction. Moreover, the pharmacological interaction is considered to be additive according to the BI theory if the observed effect is similar to the expected one (i.e., ΔE is around zero). This last interaction type is called Bliss independence [4,51,52,54,55,68]. The UT is the result of the synthesis of four major biochemical and biophysical equations (Henderson-Hasselbalch, Hill, Michaelis-Menten, and Scatchard equations) into the median-effect equation, providing the concept of CI. It is represented by Equation (2):

(2) $$\mathrm{CI}=\frac{\left(D\right)1}{\left(\mathrm{Dx}\right)1}+\frac{\left(D\right)2}{\left(\mathrm{Dx}\right)2}$$

where CI is the combination index, (Dx) is the dose of a drug alone giving an effect of x%, D is the dose of the drug in a combination giving an effect of x%, and 1 and 2 define the drugs included in the combination. CI values < 1, =1, and >1 indicate synergism, additivity, and antagonism, respectively. In other words, the smaller the CI value, the greater the magnitude of the interaction [4,46,54]. In this study, CompuSyn^®^ computer software (ComboSyn Inc., Paramus, NJ, USA) was used to calculate the CI values. The experimental data points entered into the computer software are usually obtained from the average relaxation values of multiple replicates [69]. This software also allows the calculation of the dose reduction index (DRI) for combination data points. The DRI is defined as a measure of by how many times the dose of each drug in a combination may be reduced at a given effect level, compared with the doses of each drug alone. It can be calculated using the Equation (3):

(3) $$\mathrm{DRI}=\frac{\left(D\right)1}{\left(\mathrm{Dx}\right)1}$$

A DRI value higher than 1 indicates a favorable dose reduction. The greater the value, the greater the dose reduction for a therapeutic effect. This value provides additional information on the possibility of reducing the drug dose independently of the type of interaction [46].

### 4.6. Statistical Analysis

For each studied combination, values are represented as mean ± standard error of the mean (SEM) of *n* = 5–6 different subjects. The statistical significance at each dose level was assessed by the one-way analysis of variance (ANOVA) with a Bonferroni correction. The level of statistical significance was defined as *p* < 0.05. All data analyses were performed using GraphPad Prism 5 computer software (San Diego, CA, USA).

Each BI theory analysis was completed by the calculation of a 95% confidence interval. The latter delimits the extreme values that could be expected in 95% of cases if the experiment is repeated [70]. This calculation was done using Equation (4):

(4) $$µ\;\pm 1.96\;\sqrt{\frac{\sigma^{2}}{n\;}}$$

where µ is the mean ΔE, σ is the standard deviation, and n is the number of different subjects.

In the following studies, the ΔE must be positive with a positive 95% confidence interval to conclude that there is a synergistic pharmacological interaction. If the 95% confidence interval associated with the ΔE calculation includes zero, Bliss independence is concluded according to the BI theory [68].

## 5. Conclusions

The ex vivo model of guinea pig tracheal rings represents a valuable tool for highlighting appropriate synergistic dose ratios of LABA/LAMA combinations by pharmacological identification and characterization of interactions between both classes of bronchodilator. Their impact on the contractile tone of guinea pig tracheal rings was assessed with a more specific focus on the dose ratio of LABA/LAMA (*w/w*) that promoted an optimized relaxation of the ASM. In the context of this study, findings indicate that synergy was found for both FOR/TIO and IND/TIO combinations, but not at all dose ratios. Only a well-balanced dose ratio between both classes of bronchodilator drugs allowed them to interact synergistically. In addition, airway relaxation was improved with the LABA/LAMA combination at lower concentrations than with monotherapies, which is predictive of improved clinical safety. The use of distinct mathematical models such as BI analysis and UT analysis for the pharmacological assessment of these combinations limits the risk of potentially mis-estimating pharmacological interactions. FOR/TIO 2:1 (*w/w*), FOR/TIO 3:1 (*w/w*), IND/TIO 10:1 (*w/w*), and IND/TIO 20:1 (*w/w*) could all be further investigated as their synergistic relaxant effect are not significantly different from each other. Nevertheless, FOR/TIO 2:1 (*w/w*) and IND/TIO 10:1 (*w/w*) were highlighted as the most relevant for this study because of the slightly greater magnitude of synergy. Similarly, FOR/TIO 2:1 (*w/w*) was selected to be further studied as it showed the strongest synergy and the best dose reduction compared to the same drugs as monotherapy, although not significant. This dose ratio now requires confirmation on a preclinical model of asthma and COPD by measuring lung function from bronchodilation before proceeding to clinical trials.

## Figures and Tables

**Table 1:** Relaxant effect, CI and magnitude of pharmacological interaction between FOR and TIO (A and B) and between IND and TIO (C and D) calculated using UT analysis in guinea pig tracheal rings.

**A**	**Tiotropium bromide monohydrate**	**Formoterol fumarate dihydrate**	**Relaxant effect (%)**	**CI**	**Interaction magnitude**
	1.55 nM	0.45 nM	35.12	3.522	− − − − −
	1.55 nM	0.90 nM	69.49	0.834	+ +
	1.55 nM	1.81 nM	86.51	0.294	+ + + +
	1.55 nM	2.71 nM	95.34	0.102	+ + + +
	1.55 nM	3.62 nM	98.64	0.035	+ + + + +
	1.55 nM	7.24 nM	99.3	0.020	+ + + + +
	1.55 nM	9.04 nM	99.7	0.009	+ + + + +
**B**	**Tiotropium bromide monohydrate**	**Formoterol fumarate dihydrate**	**Relaxant effect (%)**	**CI**	**Interaction magnitude**
	0.41 nM	0.12 nM	10.49	2.158	− − − −
	0.61 nM	0.35 nM	33.20	1.174	− −
	0.81 nM	0.94 nM	59.17	0.652	+ + +
	1.02 nM	1.79 nM	79.21	0.337	+ + +
	1.55 nM	3.62 nM	91.34	0.183	+ + + +
	2.08 nM	9.69 nM	97.35	0.080	+ + + + +
	2.60 nM	15.19 nM	99.59	0.060	+ + + + +
**C**	**Tiotropium bromide monohydrate**	**Indacaterol maleate**	**Relaxant effect (%)**	**CI**	**Interaction magnitude**
	1.55 nM	1.50 nM	36445,00	3.865	− − − − −
	1.55 nM	2.99 nM	69.54	0.928	+
	1.55 nM	7.48 nM	83.93	0.385	+ + +
	1.55 nM	14.96 nM	91.03	0.190	+ + + +
	1.55 nM	29.91 nM	96.59	0.076	+ + + + +
	1.55 nM	59.82 nM	99.06	0.025	+ + + + +
	1.55 nM	74.78 nM	99.64	0.011	+ + + + +
	1.55 nM	0.15 mM	99.86	0.005	+ + + + +
**D**	**Tiotropium bromide monohydrate**	**Indacaterol maleate**	**Relaxant effect (%)**	**CI**	**Interaction magnitude**
	0.41 nM	0.39 nM	9.92	18.707	− − − − − −
	0.61 nM	1.17 nM	27.91	1.779	− − − −
	0.81 nM	3.90 nM	48.10	0.895	+
	1.02 nM	9.88 nM	71.56	0.440	+ + +
	1.55 nM	29.91 nM	90.30	0.203	+ + + +
	2.08 nM	80.14 nM	97.99	0.067	+ + + + +
	2.60 nM	0.13 mM	99.12	0.041	+ + + + +
	3.13 nM	0.30 mM	99,59	0.025	+ + + + +

The smaller the CI value, the greater the magnitude of the interaction. − − − − − − Very strong antagonism; − − − − − Strong antagonism; − − − − Antagonism; − − − Moderate antagonism; − − Slight antagonism; − Nearly additive; + Slight synergism; + + Moderate synergism; + + + Synergism; + + + + Strong synergism; + + + + + Very strong synergism. CI: Combination Index.

**Table 2:** DRI values for TIO, FOR and IND in combination at concentrations inducing 50% of relaxation. DRI50: Dose reduction index at 50% of relaxation.

DRI50			
LABA/LAMA Combination	TIO	FOR	IND
FOR/TIO 2:1 (*w*/*w*)	1.38	4 338	/
FOR/TIO 3:1 (*w*/*w*)	1.23	2 586	/
IND/TIO 10:1 (*w*/*w*)	1.27	/	4 484
IND/TIO 20:1 (*w*/*w*)	1.35	/	2 370

## Data Availability

Data is contained within the article.

## Supplementary Materials

The following supporting information can be downloaded at: https://www.mdpi.com/article/10.3390/ph15080963/s1, Table S1: Percentages of tracheal relaxation obtained with increasing percentages of the vehicle dimethyl sulfoxide.

## Abbreviations

ANOVA	Analysis of variance
ASM	Airway smooth muscle
BI	Bliss independence
CI	Combination index
COPD	Chronic obstructive pulmonary disease
ΔE	Delta effect
DRI	Dose reduction index
EC50-70	Submaximal effective concentration size between 50% and 70% of the maximum concentration
FOR	Formoterol fumarate dihydrate
IND	Indacaterol maleate
LABA	Long-acting β2-agonist
LAMA	Long-acting muscarinic antagonist
pEC50	Negative logarithm of the half maximal effective concentration
SEM	Standard error of the mean
TIO	Tiotropium bromide monohydrate
UT	Unified theory
