# Supplementary material for: Optimization of Long-Acting Bronchodilator Dose Ratios Using Isolated Guinea Pig Tracheal Rings for Synergistic Combination Therapy in Asthma and COPD
Source: Pharmaceuticals (Basel). 2022 Aug 3;15(8):963. doi: 10.3390/ph15080963 (PMC9416144; doi:10.3390/ph15080963)
Supplement: Supplementary file 1 [file pharmaceuticals-15-00963-s001.zip › pharmaceuticals-1789435-supplementary.pdf]

pharmaceuticals

Supplementary Material

Optimization of long-acting bronchodilator dose ratios using isolated guinea pig tracheal rings for synergistic combination therapy in asthma and COPD

Table S1: Percentages of tracheal relaxation obtained with increasing percentages of the vehicle DMSO.

Relaxation (%E_{max} of 1 mM atropine on contraction by 10 µM MCh)								
**DMSO (%)**	0.00000025	0.0000025	0.000025	0.00025	0.00075	0.0025	0.025	0.25
**Mean relaxation (%)**	2	6	9	13	6	18	21	21
**SD (%)**	2	3	6	7	8	8	9	9
**SEM (%)**	1	1	2	3	3	3	4	4
**NB**	6	6	6	6	6	6	6	6
